# Association between PhA and Physical Performance Variables in Cancer Patients

**DOI:** 10.3390/ijerph20021145

**Published:** 2023-01-09

**Authors:** Borja Gutiérrez-Santamaría, Aitor Martinez Aguirre-Betolaza, Arturo García-Álvarez, Maria Soledad Arietaleanizbeaskoa, Nere Mendizabal-Gallastegui, Gonzalo Grandes, Arkaitz Castañeda-Babarro, Aitor Coca

**Affiliations:** 1 Department of Physical Activity and Sport Sciences, Faculty of Education and Sport, University of Deusto, 48007 Bilbao, Biscay, Spain; 2 Primary Care Research Unit of Bizkaia, Biocruces Bizkaia Health Research Institute, Plaza de Cruces 12, 48903 Barakaldo, Biscay, Spain; 3 Department of Physical Activity and Sports Sciences, Faculty of Health Sciences, Euneiz University, 01013 Vitoria-Gasteiz, Araba, Spain

**Keywords:** phase angle, bioimpedance, physical performance, cancer

## Abstract

Maintaining the physical performance of cancer patients is increasingly considered due to the growing number of cancer patients and the aggressiveness of the treatments. For this reason, bioimpedance is now being used to record patients’ body composition by obtaining the phase angle (PhA). Although there is a direct relationship between PhA, age, sex and disease prognosis, it has not been measured as an analysis of physical performance in oncology patients and is a valid tool in the follow-up of cancer patients. For this purpose, 311 patients were evaluated, and both bioelectrical impedance analysis (BIA) and physical performance measurements were performed. The modification of the results concerning PhA was found to be highly relational, as a variation in one of the variables affected the other. It was concluded that each degree increase in PhA modified −22.57 s [−27.58; −17.53] in 400-m walking test (400 mWT); 13.25 kg [10.13; 16.35] in upper-body strength (UBS); 6.3 [4.95; 7.65] in lower-body strength (LBS); 1.55 mL/kg/min [0.98; 2.12] in VO_2peak_; 6.53 Watts [3.83; 9.20] in ventilatory threshold 1 (VT1); 10.36 Watts [7.02; 13.64] in ventilatory threshold 2 (VT2). It was also noted that age was a factor that affected the relationship between PhA and 400 mWT; the older the age, the higher the relationship. PhA data has been shown to be highly correlated with physical performance. This is of great importance in clinical practice because a cancer patient’s physical performance levels can be assessed during treatment.

## 1. Introduction

The assessment of body composition changes and nutritional status using bioelectrical impedance analysis (BIA) has gained popularity, and one of the parameters measured, the phase angle (PhA), is considered an indicator of cellular health and integrity, and has been used as a prognostic marker in diseases such as cancer, HIV and other comorbidities [1].

PhA is obtained from direct measurements of the BIA, comprising resistance (in ohms), reactance (in ohms) and the derived phase angle (in degrees). The phase angle is calculated as the arc tangent of the bioelectrical impedance, reactance and resistance analysis vectors. These describe the positions of cell membranes, in the case of reactance, with body fluids, in the case of resistance, against an injected alternating electric current. Therefore, PhA is a value obtained from the raw data of the BIA analysis, making it high quality and usable data.

PhA has recently been identified as a predictor of competitive level and has been related to sports performance in athletes because it assesses the quality of cells (in terms of cell membrane improvement because it is the main aspect measured by PhA) directly related to physical performance [2,3]. It has been suggested that since increased PhA can be explained by increased muscle cell volume by intracellular fluid, it may be more sensitive for detecting muscle size and function than lean soft tissue estimates [4]. However, it is not yet known whether PhA can be used as an indicator of muscle quantity and strength and maximal aerobic power in the adult population, who also represent an understudied population, as is the case with cancer-affected populations. Skeletal muscle mass and strength, as well as aerobic fitness level, are relevant fitness parameters underlying physical performance [4].

Although physical performance is not looked for in cancer patients as it can be looked for in athletes, it is necessary to optimize the physical performance of patients to improve both their quality of life and their disease [5,6]. Physical performance in cancer patients is understood as a person’s ability to perform activities that require physical actions, ranging from self-care (activities of daily living) to more complex activities that require a combination of skills, often with a social component or within a social context [7].

Previous articles have shown that subjects with lower PhA also had reduced levels of strength and muscle mass, and were even being diagnosed with sarcopenia depending on the severity [8].

Although the evidence on PhA is still limited, it is beginning to be related to physical parameters in different pathologies. In previous articles, the authors hypothesized the relationship of phase angle with cardiovascular values. Phase angle has also been found to be related to maximal strength and fat percentage in adults with obesity [9], as well as with predicting the risk of falls in older adults [10] and type 2 diabetes patients, predicting muscle catabolism [11].

Therefore, and considering the above, this article aims to: (1) evaluate the relationship between PhA and physical fitness; (2) and determine how age and sex affect the relationship between PhA and physical performance in cancer patients. 

## 2. Methods

### 2.1. Participants

This study was a descriptive cross-sectional study of a convenience sample of patients diagnosed with cancer. Participants were referred by their oncologists or hematologists at the Cruces, Basurto and Galdakao University Hospitals in Bizkaia/Biscay, Basque Country, Spain, as part of the main project called Bizi Orain for which the protocol was published [12]. Bizi Orain is an evidence-based exercise program that adheres to the American College of Sports Medicine guidelines for cancer survivors [13] and is based on the “Life Now” exercise program for people with cancer delivered in Australia [14]. The program is administered by the Primary Care Research Unit of the Bizkaia-Biocruces Bizkaia Research Institute and the University of Deusto, and is delivered in a network of health centers equipped with Bizi Orain exercise laboratories integrated into the public health system of the Basque Country (Osakidetza).

### 2.2. Procedure

Patients were physically and psychologically evaluated at the University of Deusto, (Vizcaya, Spain) from July 2019 to July 2022. Measurements were taken at the time of study enrolment and after 3, 6 and 12 months at the same times of the day (9:00 to 14:00) and under similar environmental conditions (temperature, ±21 °C; relative humidity, 50–55%; barometric pressure, ±720 mmHg).

### 2.3. Measurements

The patient physical test evaluation begins with the measurement of body composition, followed by the evaluation of the 400 mWT, and finally, an upper and lower body strength test.

The subject’s height was measured using a wall stadiometer (Seca, Hamburg, Germany) and body composition with an Inbody 770 bioimpedance analyzer (In-body, Seoul, Korea), conforming with the measurement protocol [15]. Measurements were taken in a standing position because the position interferes with some of the values from which the PhA is taken [16].

The 400-m walk protocols consisted of a 400-m fast-paced walk administered by trained and certified personnel. The walk was conducted in a long corridor with cones at both ends, separated by 20 m [12,17].

To determine the VO_2peak_ (the maximum VO_2_ value in the last seconds of the last stage of the submaximal test performed), a test was performed on a cycloergometer with an electric brake (Ergostik, Geratherm Respiratory, Bad Kissingen, Germany). After a 5-min warm-up with no load, the load was increased by 10 W per minute from an initial load of 20 W. Participants were instructed to maintain a cadence above 65 rpm. Gas exchange was analyzed throughout the test with a gas analyzer (Ergostik, Sanro, Spain). The first and second ventilatory thresholds (VT1 and VT2) were obtained by the first exponential increase in ventilatory oxygen (O_2_) equivalent (VE/VO_2_). VT2, or respiratory compensation point (RCP), was banished using the ventilatory equivalent ratio (VE/VCO_2_) method. The test was performed until confirmation of at least one of the following criteria: (1) The second ventilatory threshold or the so-called “respiratory compensation point” (RCP) is observed from the Wasserman figures (respiratory equivalents, and O_2_ and CO_2_ partial pressure changes); (2) The respiration exchange ratio (RER) ≥ 1.05 and rating of perceived effort (RPE) > 8 on the 0–10-point Borg scale; 3) Participants exhibit volitional exhaustion without meeting the previous criteria [18,19]. 

As the assessment and measurement of VO_2Max_ is the most accurate value to know the state of the cardiovascular system and the implication in the prediction of mortality because of any cause [20], it is an expensive and laborious method. For this reason, correlations were sought between this value and others of greater complexity and price at the time of assessment. For example, long-distance walking tests, such as the 400-m walking test (400 mWT), when performed as fast as possible, are widely used to assess cardiorespiratory fitness instead of the VO_2Max_ test [21].

Through the test of five maximum repetitions (5RM) and using strength exercise machines, general muscular strength was evaluated with chest press exercises (L070, BH, Vitoria, Spain) and leg press (L050, BH, Vitoria, Spain) [22]. 

### 2.4. Statistical Analysis

The relationship between phase angle and the physical tests was analyzed using mixed linear regression models (SAS: PROC MIXED), which account for the correlations between repeated measurements for each patient. Age and sex were used as adjustment variables, and the best model was chosen following a stepwise backward strategy using likelihood ratio tests (with a significance criterion of *p* < 0.05). We also performed correlation tests between the PhA and the physical variables.

Subgroup analyses were carried out to study whether the relationship between PhA and the outcome variables was different depending on sex or age, testing interaction terms between these covariates and angle phase (*p* < 0.01). All analyses were performed with SAS 9.4 (SAS Institue Inc., Cary, NC, USA) and R.4.2.2 (The R Foundation, Vienna, Austria).

## 3. Results

Of the 311 patients included in the study, 309 had at least one PhA measurement, 239 had at least two, 201 had at least three and 94 had four measurements, respectively. Therefore, the total data were 843 PhA measurements. Table 1 shows the characteristics of the sample according to the main descriptive variables.

**Figure 1:**
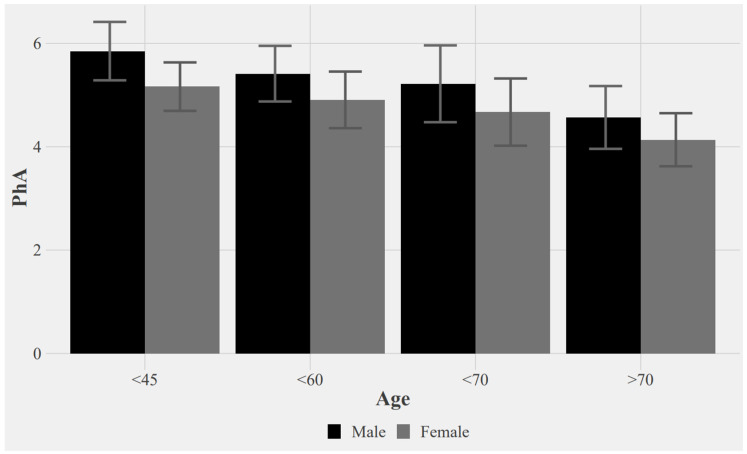
PhA values according to the age and sex of subjects.

Firstly, and according to Figure 1, a descending pattern of the phase angle in relation to age is observed in both sexes, being higher in men with respect to women in all age ranges.

Regarding the relationship values shown in Table 2, a statistically significant relationship is observed when comparing the PhA value against the 400 mWT having a reduction of 22.57 s (CI = −27.58 to −17.53) for each one-degree increase in the PhA.

Looking at the behavior of PhA compared to strength, we can see how the comparison of PhA with both upper and lower body strength is statistically significant. We see an increase of 13.25 kg (CI = 10.13 to 16.35) in lower body strength for each one-degree increase in PhA. Likewise, an increase of 6.3 kg (CI = 4.95 to 7.65) in lower body strength for each grade increase in PhA.

When observing the behavior of PhA compared to VO_2peak_, we can determine that there is a significant comparison between the two values observing an increase of 1.55 mL/kg/min (CI = 0.98 to 2.12) for each one-degree increase in PhA. Similarly, watts (W) increases of 6.53 W (CI = 3.83 to 9.2) and 10.36 W (CI = 7.02 to 13.64) are also shown in VT1 and VT2, respectively, for each one-degree increase in PhA.

Model results, including the effect of sex and age, are shown in Supplementary Table S1.

**Figure 2:**
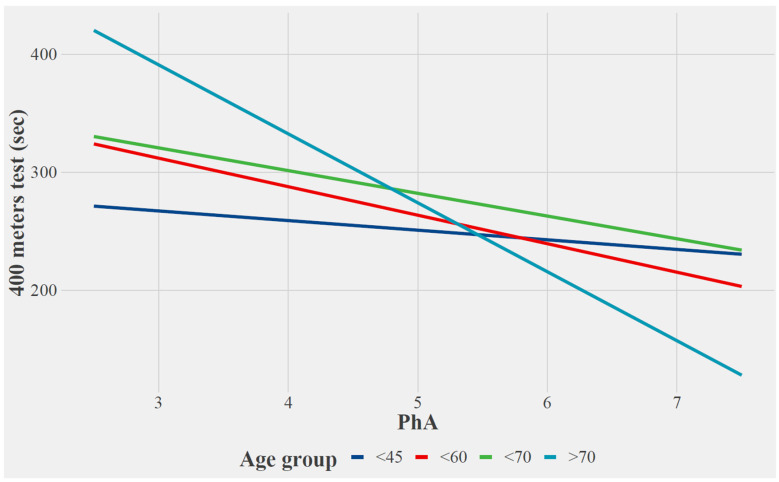
Estimated relationship between PhA and 400 mWT by age group.

The effect of phase angle on any of the variables was not significantly modified by age or sex except the 400 mWT by age. Estimates from this model can be observed in Figure 2; although, for all patients, an increase in PhA implies a decrease in the time required to perform the 400 mWT, and this decrease is significantly greater for the older group of patients. Results for this model and the rest of the subgroup analysis are shown in Supplementary Table S2.

## 4. Discussion

In this research, PhA was used as a predictor of physical performance, considering that it is a non-invasive, cheap and fast method with a high correlation with 400 m WT, UBS, LBS, VO_2_ peak, VT1 and VT2. Thus, it fulfills the objectives of studying the relationship between PhA values and physical performance, and analyzing if this relationship is affected by differences in age and sex.

A high PhA represents good cell quality (from the point of view of the cell membrane because it is the parameter analyzed by PhA), and a low PhA represents a decreased cell quality or even a cell death. PhA usually ranges from 4 to 9 in healthy individuals and decreases in females, as well as with increasing age, lower for females, high body mass index and various disease states, such as cancer [23]. In our study, as in previous research, this pattern of change in PhA according to age and sex has been observed.

The mean PhA of our sample is 4.88°, being lower than the values collected in a healthy reference population with normal BMI (18.5–25) and mean age between 50–59 years (referring to the mean age of our sample) that showed a PhA of 5.73 ± 0.68, about one degree higher than the cancer sample studied. Moreover, our sample, even has lower PhA than healthy people with obesity (BMI > 40) aged between 50–59 years (referring to the mean age of our sample) with a PhA of 5.81± 0.7 and even people older than 70 years with BMI > 40 with PhA mean of 5.07 ± 0.72 [24].

Sex and age were the main determinants of PhA in adults, with males and younger subjects having higher PhA [24]. Other studies show similar results in adults. The phase angle was significantly lower in women than in men and was lower at older ages [25]. As can be seen in Figure 1 and Figure 2, our study sample behaves in the same way, which corroborates the results of previous research on the change in PhA according to age and sex. This is an important characteristic to consider when evaluating the PhA value. Due to these results obtained in other research studies, and as our sample behaves in the same way, it was proposed to analyze the data, considering these two variables of important relevance for the interpretation of the data.

Continuing with the functional capacity assessments, it has been observed in other studies that breast cancer patients, divided into two groups by PhA ≤ 5.6 or >5.6 degrees, do not show significant differences in the 6 min walking test (*p* = 0.678) [26]. This research was carried out in a small population sample of *n* = 25 divided into two groups of *n* = 12 men and *n* = 13 women, much smaller than the sample of our study (*n* = 311). Specifically, in our research, considering the results of 311 cancer patients, we can observe that the 400 mWT values can be predicted with great accuracy and significance by measuring the PhA, so it could be a useful tool to predict the improvement of functional capacity in a clinical setting where it may not be possible to perform functional capacity measurements such as the 6 min walking test or the 400 mWT. In these results, two different tests are being compared (6-min walking test and 400 mWT), but, due to lack of scientific evidence on the relationship of PhA and 400 mWT, and taking into account that the nature of the tests is similar, we accept the comparison without the same test being used in each case [27].

On the other hand, a reduction in muscle mass and strength is part of the symptomatology of sarcopenia, even more in cancer patients [28]. Previous review articles have shown a relationship between phase angle and sarcopenia [8]. It shows how PhA is decreased in sarcopenic subjects leading to reduced strength in these patients. It was also evidenced that the prevalence of sarcopenia is higher in subjects with low PhA, although further studies are needed to determine to what extent PhA may be valuable in detecting low muscle quality and/or identifying sarcopenia [8]. In our study we have seen increases in strength levels as PhA increases. This shows how the strength value is strongly related to PhA and is, therefore, a parameter that provides information on strength levels without the need for a specific strength test, which facilitates the follow-up of the cancer patient. Many studies have reported the clinical relevance of decreased phase angle or sarcopenia as a predictor of shorter cancer survival [29,30,31,32]. Given the importance of phase angle as a predictor of sarcopenia and poor muscle quality, our finding of the association of PhA with an increase in both upper and lower body strength levels may be a key element in improving quality of life. If this increase in strength is maintained over time, it will lead to an increase in muscle mass and a reversal or delay in the onset of sarcopenia.

It is well known that when these events occur, the prognosis against any disease is worse, and this has an important clinical relevance since knowing this relationship. Special attention can be paid to patients at risk of sarcopenia to reduce, slow down and even reverse the disease to improve the patient’s functionality, and thus their prognosis against the disease, since sarcopenia is an adverse effector in cancer patients. This is associated with reduced performance status, complications, and overall survival [33,34]. It has also been observed that as a result of the treatments, patients’ nutrition worsens and leads to cases of sarcopenia. Before treatment, 69.1% of patients were well-nourished, 16.4% were malnourished, and 14.5% were cachectic/sarcopenic; post-treatment proportions were 16.4%, 45.4% and 38.2%, respectively [32]. Therefore, cancer patients should pay particular attention to nutrition, especially protein levels consumed [35], and to specific physical exercise aimed at building or maintaining muscle mass in order to increase strength levels [36] and thus PhA.

Regarding the VO_2peak_ values (which represent the gold standard in the representation of the level of functional capacity), the significance shown by the value when predicted by the PhA can be observed. The observed values appear not to be significantly relevant due to their small magnitude (1.55 each PhA angle), but it can be determined that slight increases in patients with a low VO_2peak_ (mean of 15.3 ± 4) may be significant as a health improvement. The slight beneficial changes observed in VO_2peak_ are clinically relevant because VO_2peak_ is an important predictor of all-cause mortality [37,38]. Our results, combined with previous findings of VO_2peak_ impairment among cancer patients with observed values ranging from 15.75 ± 5.52 to 29.82 ± 5.08 [39,40,41,42,43], emphasize the clinical importance of increasing or maintaining VO_2peak_ at this stage of the cancer trajectory. Therefore, although changes in VO_2peak_ that in the normal population would be slightly low (changes of 1.55 per each degree of PhA) are observed, in cancer patients, they could be decisive in maintaining functionality in activities of daily living

VO_2peak_ alone only represents the organism’s capacity to capture, transport and consume oxygen during exercise [44]. It is, therefore advisable to also observe the improvement in VT1 and VT2 since these are the thresholds at which modifications in the individual’s physiology occur and are used to prescribe physical exercise in athletes [45] and in cancer patients [46]. Therefore, the significant relationship between increases in VT1 and VT2 with PhA makes it a predictor of improvement in both VO_2peak_ and VT1 and VT2, representing an improvement in the functional capacity of cancer patients. As the assessment and measurement of VO_2Max_ is the most accurate value to know the state of the cardiovascular system and the implication in the prediction of mortality because of any cause [37], it is an expensive and laborious method, which is why sometimes, this methodology is unavailable. For this reason, correlations were sought between this value and others of greater complexity and price at the time of assessment. Long-distance walk tests like the 400 mWT, when performed as fast as possible, are widely used to assess cardiorespiratory fitness [47]. Through this study, we are giving another alternative to expensive laboratory VO_2peak_ tests, by means of PhA knowing that they are closely related, we could know, with its limitations in terms of accuracy, at what VO_2peak_ levels the cancer patient could be at, knowing that if PhA values increase, most likely their VO_2peak_ values will also increase.

As for the relationship between 400 mWT performance, PhA and age, it can be observed that the older the age, the greater the relationship between these variables. This may be because the 400 mWT is used at older ages to determine the function of that older person [48]. Therefore, what we can determine, is that the older, the better the relationship between PhA and 400 mWT. That is why it is an important fact to consider when making this prediction of performance.

## 5. Conclusions

BIA is a popular method because it is non-invasive, inexpensive and fast for monitoring changes in body composition in cancer patients.

It has been shown that PhA data directly obtained from the BIA test is highly related to physical performance, and thus provides information on patient changes during treatment. In particular, the relationship with the 400 mWT has been shown to be a very valid test for predicting sarcopenia problems and estimating the VO_2Max_ of patients, especially in older adults, and even to be related to death from any cause. This is of great importance in clinical practice because, without the need to carry out long and costly laboratory tests, the physical performance levels of a cancer patient during treatment can be assessed

## 6. Future Research Lines

More research is needed to confirm the hypothesis, and it would be convenient to know PhA values in which the oncologist would have information that could be a red flag and be able to pay special attention to this patient to maintain the physical performance in order to not deteriorate his quality of life.

## 7. Limitations

It should be known that there is evidence that the phase angle measurement can vary according to the measuring device and body position at the time of measurement, being different to measure standing to supine [16]. During the research carried out in this investigation, all the measurements were made in a standing position, so that any other position could not recreate the same results.

## Figures and Tables

**Table 1:** Characteristics of the sample. Data is shown as mean ± standard deviation (SD) or percentage (%).

Variable	*n* (%)	Mean ± SD
Age (years)	311 (100)	55.54 ± 10.98
Sex		
Female Male	225 (712.3) 86 (27.7)	
Height (cm)		164.7 ± 8.7
Weight (kg)		70.93 ± 14.52
BMI (kg/m^2^)		26.1 ± 4.9
PhA (°)		4.88 ± 0.68
400 mWT (seg.)		278.73 ± 53.37
VO_2peak_ (mL/kg/min)		15.31 ± 4
VT1		54.09 ± 16.81
VT2		80.97 ± 23.17
UBS		61.70 ± 23.17
LBS		33.26 ± 13.37
Surgery	164 (52.9)	
Metastatic	50 (19.6)	
Cancer stage		
I II III IV	48 (15.4) 80 (25.7) 62 (19.9) 121 (38.9)	

BMI: body mass index; PhA: phase angle; 400 mWT: 400 m walking test; VO_2peak_: peak consumption of VO_2_; VT1: first ventilatory threshold; VT2: second ventilatory threshold; UBS: upper-body strength; LBS: Lower-Body strength.

**Table 2:** Relationship of physical performance and the PhA.

Variable	Mixed Linear Regression Estimate [95% CI]	Mixed Linear Regression *p*-Value	Pearson Correlation [95% CI]	*p*-Value
400 mWT (sec)	−22.57 [−27.58; −17.53]	<0.001	−0.36 [−0.42; −0.3]	<0.001
UBS (kg)	6.3 [4.95; 7.65]	<0.001	0.54 [0.48; 0.59]	<0.001
LBS (kg)	13.25 [10.13; 16.35]	<0.001	0.47 [0.42; 0.53]	<0.001
VO_2peak_ (mL/kg/min)	1.55 [0.98; 2.12]	<0.001	0.25 [0.18; 0.32]	<0.001
VT1 (W)	6.53 [3.83; 9.20]	<0.001	0.35 [0.28; 0.42]	<0.001
VT2 (W)	10.36 [7.02; 13.64]	<0.001	0.42 [0.36; 0.49]	<0.001

Sec: seconds; kg: kilograms; W: watts; UBS: upper-body strength; LBS: Lower-Body strength. Statistical significance set at *p* < 0.05.

## Data Availability

All available data can be obtained by contacting the corresponding author.

## Supplementary Materials

The following supporting information can be downloaded at: https://www.mdpi.com/article/10.3390/ijerph20021145/s1. Table S1: Relationship of physical performance and the PhA; Table S2: Subgroups analysis.
