# Supplementary material for: Association between PhA and Physical Performance Variables in Cancer Patients
Source: Int J Environ Res Public Health. 2023 Jan 9;20(2):1145. doi: 10.3390/ijerph20021145 (PMC9859119; doi:10.3390/ijerph20021145)
Supplement: Supplementary file 1 [file ijerph-20-01145-s001.zip › ijerph-2064129-supplementary.pdf]

International Journal of
Environmental Research and Public Health

MDPI

Supplementary Materials

Table S1. Relationship of physical performance and the PhA.

Variable			Estimate	95%CI	*p*-value
400mWT					
	Intercept		367.42	[338.22; 396.62]	<0.001
	PhA		−22.57	[−27.58; −17.53]	<0.001
	Age				<0.001
		<45	Ref		
		<60	9.11	[−5.34; 23.55]	
		<70	27.24	[11.63; 42.86]	
		70+	47.65	[26.77; 68.53]	
UBS					
	Intercept		18.83	[10.59; 27.08]	<0.001
	PhA		6.3	[4.95; 7.65]	<0.001
	Age				0.005
		<45	Ref		
		<60	−1.51	[−4.09; 1.06]	
		<70	−4.07	[−6.93; −1.2]	
		70+	−5.62	[−9.49; −1.74]	
	Sex				<0.001
		Male	Ref		
		Female	−17.76	[−19.89; −15.64]	
LBS					
	Intercept		18.52	[−0.5; 37.54]	0.057
	PhA		13.25	[10.13; 16.35]	<0.001
	Age				0.047
		<45	Ref		
		<60	−0.98	[−6.92; 4.95]	
		<70	−6.46	[−13.11; 0.18]	
		70+	−8.99	[−18.01; 0.03]	
	Sex				<0.001
		Male	Ref		
		Female	−17.41	[−22.42; −12.4]	
VO2 peak					
	Intercept		8.95	[5.8; 12.11]	<0.001
	PhA		1.55	[0.98; 2.12]	<0.001
	Age				0.012
		<45	Ref		
		<60	−0.57	[−1.75; 0.61]	
		<70	−1.93	[−3.23; −0.63]	
		70+	−1.59	[−3.52; 0.34]	
VT1					
	Intercept		40.79	[24.45; 57.12]	<0.001
	PhA		6.53	[3.83; 9.2]	<0.001

	Age				<0.001
		<45	Ref		
		<60	−2.79	[−7.66; 2.08]	
		<70	−11.77	[−17.37; −6.18]	
		70+	−10.42	[−19.01; −1.82]	
	Sex				<0.001
		Male	Ref		
		Female	−15.04	[−19.41; −10.67]	
VT2					
	Intercept		57.21	[36.95; 77.46]	<0.001
	PhA		10.36	[7.02; 13.64]	<0.001
	Age				<0.001
		<45	Ref		
		<60	−3.71	[−10; 2.57]	
		<70	−17.85	[−25.04; −10.65]	
		70+	−15.58	[−26.61; −4.54]	
	Sex				<0.001
		Male	Ref		
		Female	−22.59	[−28.2; −16.98]	

Table S2. Subgroups analysis.

Subgroup Analysis			Estimate	95%CI	*p*-value
400mWT, Age					
	Intercept		291.67	[220.67; 362.66]	<0.001
	PhA		−8.15	[−21.46; 5.16]	<0.001
	Age				<0.001
		<45	Ref		
		<60	92.74	[12.97; 172.51]	
		<70	86.78	[3.05; 170.5]	
		70+	274.66	[164.02; 385.31]	
	PhA*Age				<0.001
		PhA*<45	Ref		
		PhA*<60	−16	[−31.12; −0.89]	
		PhA*<70	−11.11	[−27.11; 4.89]	
		PhA*70+	−50.29	[−74.13; −26.44]	
400mWT, Sex					
	Intercept		381.34	[333.48; 429.21]	<0.001
	PhA		−24.15	[−32.54; −15.76]	<0.001
	Sex				0.275
		Male	Ref		
		Female	−13.6	[−67.25; 40.05]	
	Age				<0.001
		<45	Ref		
		<60	8.44	[−6.09; 22.97]	
		<70	25.16	[9.04; 41.27]	
		70+	45.3	[23.99; 66.61]	
	PhA*Sex				0.791
		PhA*Male	Ref		
		PhA*Female	1.39	[−8.92; 11.71]	
UBS, Age					
	Intercept		3.05	[−17.6; 23.7]	0.772
	PhA		9.32	[5.45; 13.18]	<0.001
	Sex				<0.001
		Male	Ref		
		Female	−17.8	[−19.92; −15.69]	
	Age				0.005
		<45	Ref		
		<60	12.9	[−9.66; 35.46]	
		<70	19.15	[−3.49; 41.79]	
		70+	2.9	[−24.88; 30.68]	
	PhA*Age				0.16
		PhA*<45	Ref		
		PhA*<60	−2.74	[−7.05; 1.57]	
		PhA*<70	−4.53	[−8.87; −0.183]	
		PhA*70+	−1.29	[−7.16; 4.57]	
UBS, Sex					
	Intercept		9.2	[−2.55; 20.96]	0.125
	PhA		8.18	[6.06; 10.31]	<0.001

	Sex				<0.001
		Male	Ref		
		Female	−2.77	[−16.07; 10.53]	
	Age				0.003
		<45	Ref		
		<60	−1.78	[−4.34; 0.78]	
		<70	−4.38	[−7.23; −1.53]	
		70+	−5.86	[−9.7; −2.02]	
	PhA*Sex				0.025
		PhA*Male	Ref		
		PhA*Female	−2.95	[−5.53; −0.37]	
LBS, Age					
	Intercept		23.05	[−20.79; 66.89]	0.303
	PhA		12.41	[4.26; 20.56]	<0.001
	Sex				<0.001
		Male	Ref		
		Female	−17.51	[−22.52; −12.51]	
	Age				0.046
		<45	Ref		
		<60	−6.09	[−54.36; 42.17]	
		<70	0.86	[−48.17; 49.9]	
		70+	−61.29	[−122.73; 0.15]	
	PhA*Age				0.132
		PhA*<45	Ref		
		PhA*<60	0.98	[−8.25; 10.2]	
		PhA*<70	−1.55	[−10.97; 7.86]	
		PhA*70+	12.17	[−0.94; 25.29]	
LBS, Sex					
	Intercept		23.77	[−3.93; 51.46]	0.093
	PhA		12.24	[7.27; 17.22]	<0.001
	Sex				<0.001
		Male	Ref		
		Female	−25.4	[−56.47; 5.67]	
	Age				0.049
		<45	Ref		
		<60	−0.89	[−6.84; 5.05]	
		<70	−6.35	[−13.02; 0.31]	
		70+	−8.91	[−17.94; 0.13]	
	PhA*Sex				0.61
		PhA*Male	Ref		
		PhA*Female	1.56	[−4.44; 7.56]	
VO2 peak, Age					
	Intercept		11.82	[3.79; 12.85]	0.004
	PhA		1.01	[−0.51; 2.52]	<0.001
	Age				0.011
		<45	Ref		
		<60	−2.26	[−11.33; 6.81]	
		<70	−6.38	[−15.78; 3.02]	

		70+	−9.73	[−22.35; 2.89]	
	PhA*Age				0.5
		PhA*<45	Ref		
		PhA*<60	0.31	[−1.42; 2.04]	
		PhA*<70	0.87	[−0.94; 2.68]	
		PhA*70+	1.8	[−0.95; 4.55]	
VO2 peak, Sex					
	Intercept		9.15	[3.65; 14.66]	0.001
	PhA		1.65	[0.66; 2.63]	0.001
	Sex				0.124
		Male	Ref		
		Female	1.22	[−4.96; 7.4]	
	Age				0.004
		<45	Ref		
		<60	−0.69	[−1.87; 0.49]	
		<70	−2.26	[−3.62; −0.91]	
		70+	−1.9	[−3.85; 0.05]	
	PhA*Sex				0.512
		PhA*Male	Ref		
		PhA*Female	−0.4	[−1.58; 0.79]	
VT1, Age					
	Intercept		38.41	[0.22; 76.61]	0.049
	PhA		6.98	[−0.17; 14.14]	<0.001
	Sex				<0.001
		Male	Ref		
		Female	−15.01	[−19.39; −10.62]	
	Age				<0.001
		<45	Ref		
		<60	−1.83	[−43.32; 39.65]	
		<70	−4.29	[−47.48; 38.9]	
		70+	−22.85	[−85.46; 39.75]	
	PhA*Age				0.897
		PhA*<45	Ref		
		PhA*<60	−0.17	[−8.14; 7.79]	
		PhA*<70	−1.48	[−9.81; 6.84]	
		PhA*70+	2.97	[−10.57; 16.52]	
VT1, Sex					
	Intercept		44.52	[18.6; 70.44]	<0.001
	PhA		5.83	[1.17; 10.48]	<0.001
	Sex				<0.001
		Male	Ref		
		Female	−20.35	[−49.4; 8.71]	
	Age				<0.001
		<45	Ref		
		<60	−2.73	[−7.61; 2.15]	
		<70	−11.73	[−17.33; −6.14]	
		70+	−10.34	[−18.94; −1.74]	
	PhA*Sex				0.717

		PhA*Male	Ref		
		PhA*Female	1.02	[−4.5; 6.54]	
VT2, Age					
	Intercept		57.72	[11.8; 103.63]	0.014
	PhA		10.25	[1.67; 18.83]	<0.001
	Sex				<0.001
		Male	Ref		
		Female	−22.55	[−28.17; −16.92]	
	Age				<0.001
		<45	Ref		
		<60	−10.64	[−60.84; 39.57]	
		<70	−5.66	[−58.17; 46.85]	
		70+	−37.9	[−113.88; 38.08]	
	PhA*Age				0.644
		PhA*<45	Ref		
		PhA*<60	1.38	[−8.25; 11]	
		PhA*<70	−2.46	[−12.57; 7.66]	
		PhA*70+	5.17	[−11.33; 21.68]	
VT2, Sex					
	Intercept		56.76	[24.76; 88.75]	<0.001
	PhA		10.44	[4.71; 16.17]	<0.001
	Sex				<0.001
		Male	Ref		
		Female	−21.98	[−57.82; 13.87]	
	Age				<0.001
		<45	Ref		
		<60	−3.72	[−10.03; 2.6]	
		<70	−17.85	[−25.06; −10.64]	
		70+	−15.58	[−26.64; −4.52]	
	PhA*Sex				0.973
		PhA*Male	Ref		
		PhA*Female	−0.12	[−6.92; 6.69]	
